# Supplementary material for: Genome-Wide Identification, Characterization, and Expression Analysis Related to Low-Temperature Stress of the CmGLP Gene Family in Cucumis melo L
Source: Int J Mol Sci. 2022 Jul 25;23(15):8190. doi: 10.3390/ijms23158190 (PMC9330424; doi:10.3390/ijms23158190)
Supplement: Supplementary file 1 [file ijms-23-08190-s001.zip › ijms-1803131-supplementary.pdf]

Supplementary Data

Table S1. Genes and sequence numbers used to construct phylogenetic trees.

Species	Gene Name	Sequence Number
Cucumber	*CsGLP1-1*	Csa1G007890.1
	CsGLP1-2	Csa1G007900.1
	CsGLP1-3	Csa1G166250.1
	CsGLP1-4	Csa1G537570.1
	CsGLP1-5	Csa1G596420.1
	CsGLP1-6	Csa1G662790.1
	CsGLP1-7	Csa1G662810.1
	CsGLP2-1	Csa2G035370.1
	CsGLP2-2	Csa2G174130.1
	CsGLP3-1	Csa3G146460.1
	CsGLP3-2	Csa3G218160.1
	CsGLP3-3	Csa3G218170.1
	CsGLP3-4	Csa3G384800.1
	CsGLP3-5	Csa3G386310.1
	CsGLP3-6	Csa3G386810.1
	CsGLP3-7	Csa3G386820.1
	CsGLP3-8	Csa3G644800.1
	CsGLP5-1	Csa5G128780.1
	CsGLP5-2	Csa5G129280.1
	CsGLP5-3	Csa5G614670.1
	CsGLP6-1	Csa6G290870.1
	CsGLP6-2	Csa6G404270.1
	CsGLP6-3	Csa6G452110.1
	CsGLP6-4	Csa6G502040.1
	CsGLP6-5	Csa6G525540.1
	CsGLP6-6	Csa6G525550.1
	CsGLP6-7	Csa6G525580.1

	CsGLP6-8	Csa6G525590.1
	CsGLP6-9	Csa6G525600.1
	CsGLP6-10	Csa6G525610.1
	CsGLP6-11	Csa6G525620.1
	CsGLP7-1	Csa7G281380.1
	CsGLP7-2	Csa7G337100.1
	CsGLP7-3	Csa7G368140.1
	CsGLP7-4	Csa7G380130.1
	CsGLP7-5	Csa7G450510.1
	CsGLP7-6	Csa7G452090.1
	CsGLPu	CsaUNG024810
Arabidopsis	*AtGLP1-1*	AT1G02335.1
	AtGLP1-2	AT1G09560.1
	AtGLP1-3	AT1G10460.1
	AtGLP1-4	AT1G18970.1
	AtGLP1-5	AT1G18980.1
	AtGLP1-6	AT1G72610.1
	AtGLP1-7	AT1G74820.1
	AtGLP3-1	AT3G04150.1
	AtGLP3-2	AT3G04170.1
	AtGLP3-3	AT3G04180.1
	AtGLP3-4	AT3G04190.1
	AtGLP3-5	AT3G04200.1
	AtGLP3-6	AT3G05930.1
	AtGLP3-7	AT3G05950.1
	AtGLP3-8	AT3G10080.1
	AtGLP3-9	AT3G62020.1
	AtGLP4-1	AT4G14630.1
	AtGLP5-1	AT5G20630.1

	AtGLP5-2	AT5G26700.1
	AtGLP5-3	AT5G38910.1
	AtGLP5-4	AT5G38930.1
	AtGLP5-5	AT5G38940.1
	AtGLP5-6	AT5G38960.1
	AtGLP5-7	AT5G39100.1
	AtGLP5-8	AT5G39110.1
	AtGLP5-9	AT5G39120.1
	AtGLP5-10	AT5G39130.1
	AtGLP5-11	AT5G39150.1
	AtGLP5-12	AT5G39160.1
	AtGLP5-13	AT5G39180.1
	AtGLP5-14	AT5G39190.1
	AtGLP5-15	AT5G61750.1
Rice	*OsGLP1-1*	LOC_Os01g14670.1
	OsGLP1-2	LOC_Os01g18170.1
	OsGLP1-3	LOC_Os01g50900.1
	OsGLP1-4	LOC_Os01g72290.1
	OsGLP1-5	LOC_Os01g72300.1
	OsGLP2-1	LOC_Os02g29000.1
	OsGLP2-2	LOC_Os02g29010.1
	OsGLP2-3	LOC_Os02g29020.1
	OsGLP2-4	LOC_Os02g32980.1
	OsGLP3-1	LOC_Os03g08150.1
	OsGLP3-2	LOC_Os03g44880.1
	OsGLP3-3	LOC_Os03g48750.1
	OsGLP3-4	LOC_Os03g48760.1
	OsGLP3-5	LOC_Os03g48770.1
	OsGLP3-6	LOC_Os03g48780.1
	OsGLP3-7	LOC_Os03g58980.1

OsGLP3-8	LOC_Os03g59010.1
OsGLP3-9	LOC_Os03g58990.1
OsGLP4-1	LOC_Os04g52720.1
OsGLP5-1	LOC_Os05g10830.1
OsGLP5-2	LOC_Os05g19670.1
OsGLP8-1	LOC_Os08g08920.1
OsGLP8-2	LOC_Os08g08960.1
OsGLP8-3	LOC_Os08g08970.1
OsGLP8-4	LOC_Os08g08980.1
OsGLP8-5	LOC_Os08g08990.1
OsGLP8-6	LOC_Os08g09000.1
OsGLP8-7	LOC_Os08g09010.1
OsGLP8-8	LOC_Os08g09020.1
OsGLP8-9	LOC_Os08g09040.1
OsGLP8-10	LOC_Os08g09060.1
OsGLP8-11	LOC_Os08g09080.1
OsGLP8-12	LOC_Os08g13440.1
OsGLP8-13	LOC_Os08g35750.1
OsGLP8-14	LOC_Os08g35760.1
OsGLP9-1	LOC_Os09g39510.1
OsGLP9-2	LOC_Os09g39520.1
OsGLP9-3	LOC_Os09g39530.1
OsGLP11-1	LOC_Os11g33110.1
OsGLP12-1	LOC_Os12g05840.1
OsGLP12-2	LOC_Os12g05860.1
OsGLP12-3	LOC_Os12g05870.1
OsGLP12-4	LOC_Os12g05880.1

Table S2. Gene-specific primers designed for qRT-PCR and dual luciferase assay.

**Gene Name**	**Forward Primer**	**Reverse Primer**

CmActin-7	CATATGTTGCTCTTGACTACGAAC	ACAACCTTAATCTTCATGCTGCTG
CmGLP1-1 (qRT)	CACTCCCTACTCATCATTTCC	GTTTCCAGCAGCACCTAAG
CmGLP2-1 (qRT)	CAGAAGTAACTGCGGATG	GGATAGCCCAAGTGTATTG
CmGLP2-2 (qRT)	CCGACCCGTTACAAGAC	CCAGGCAATTTAAGAACA
CmGLP2-3 (qRT)	GCTGGAAACACCATGAAC	CAATGCGAGCCAAAGAG
CmGLP2-4 (qRT)	CCGACCCGTTACAAGAC	GTCCAGGCAATTTAAGAAC
CmGLP2-5 (qRT)	CAGAAGTAACTGCGGATG	GGATAGCCCAAGTGTATTG
CmGLP3-1 (qRT)	CGACTTTGCTCGTTCGG	GGGAGGGATAAGGCTGATG
CmGLP4-1 (qRT)	CAACTTCGGACTTCAACTC	CAGTGACAATCTTCACCAC
CmGLP5-1 (qRT)	CTGCCTCATCATTCCTCTGC	CAGGTGAATCCGTTTACTTTG
CmGLP8-1 (qRT)	TTCTTCTTCAGTGGGTTGC	GATTTGGACCACATTCACC
CmGLP8-2 (qRT)	CCCAAGGTTGTAACTGTAGATG	TTGAGCCCAGGGATTTG
CmGLP8-3 (qRT)	GGCAGATGTCAATAATCCTGT	GAAGCCCACTCCTGAAGAA
CmGLP8-4 (qRT)	TCCGATCCTAGTCCACTTC	GCCACATTTAGCCCACTC
CmGLP8-5 (qRT)	CTTTTCTTCCTCGCATTTC	TGCCACATTCAACCCAC
CmGLP8-6 (qRT)	ACCCTTCTCGGAAACTGG	GTGCGGCGGATTTATTG
CmGLP8-7 (qRT)	GTGACAGCGGATGATTTC	CACATTCACAGGGCTCAC
CmGLP8-8 (qRT)	CCCTTCTCGGAAACTGG	GTGCGGCGGATTTATTG
CmGLP9-1 (qRT)	GCCGCCATCAACAACTC	GATTCGAGCGAGGGAGAC
CmGLP9-2 (qRT)	AGGACTTATGCGTCGCTG	GGAAATGCCGAGGGTG
CmGLP10-1 (qRT)	GCAGGCAGTTCTCATTCG	CATTCCCAGCGTGTTTAG
CmGLP12-1 (qRT)	GCTCAGCTTTCACCGATTTC	CCGGGTACTGGAGGATGG
CmGLP12-2 (qRT)	CGGTGGACTTCACATTG	GAGCGTGTTGAGTCCTG
CmGLP2-5-pGreen 0800-LUC	ctatagggcgaattgggtaccAGCAAGTTCGG ATATTTG	caggaatcgatacaagcttGGAAGTAGTTAA CAGCGTAAC
CmMYB23-pGreen 62-SK	ctatagggcgaattgggtaccATGACTGAAGA AGAAGAAGAGTCTC	caggaattcgatatcaagcttTTACAGGAAATT ACTCATCCACTC
CmWRKY33-pGreen 62-SK	ctatagggcgaattgggtaccATGGCCTCCTC TTCCGG	caggaattcgatatcaagcttTTAACATAGGAG AGATTGGATGAAC

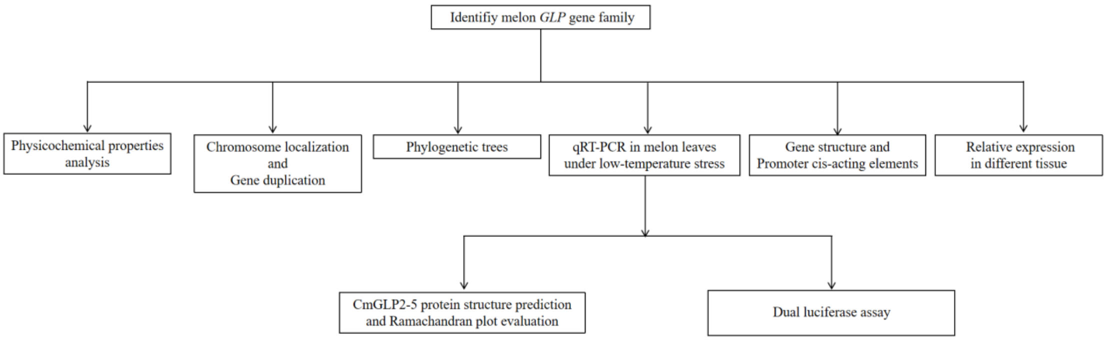

Figure S1. Flowchart of methodology.

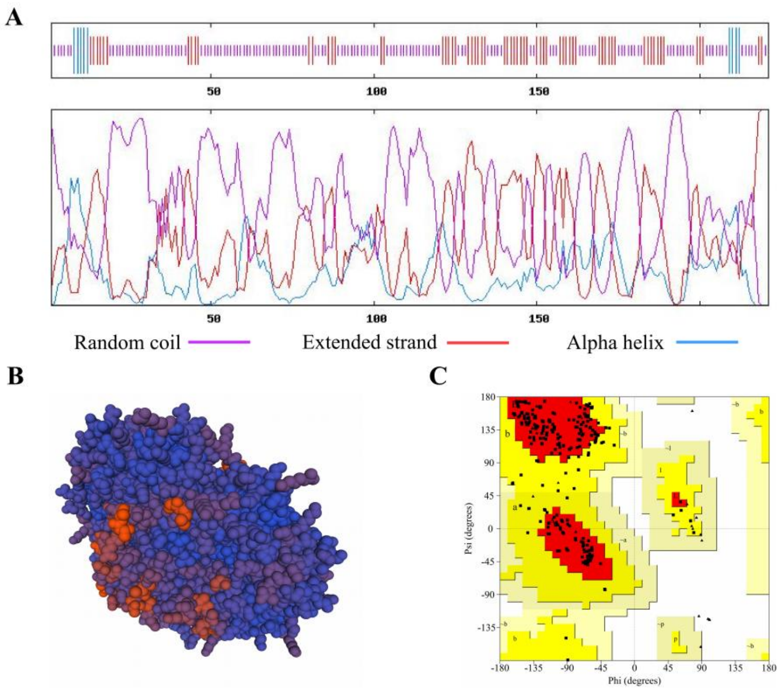

Figure S2. CmGLP2-5 protein structure prediction. **A** CmGLP2-5 protein secondary structure prediction. The purple line represents Random Coil, the red line represents Extended Strand, and the blue line represents Alpha Helix. The position of protein secondary structure can be inferred from the scale below. **B** CmGLP2-5 protein tertiary structure prediction. The color from red to blue in the protein tertiary structure prediction graph indicates the increase in the confidence of the predicted structure, and the results show that the confidence of most predicted structures is high. **C** Ramachandran plot to assess CmGLP2-5 protein tertiary structure. Ramachandran plot can classify the distribution of protein amino acid residues into four criteria: most favored regions (red regions), additional allowed regions (yellow regions), generously allowed regions (light yellow regions) and disallowed regions (white regions). All amino acid residues of CmGLP2-5 protein are distributed in most favored regions and additional allowed regions, of which 86.3% are in most favored regions and 13.7% are in additional allowed regions. Therefore, we speculate that the protein has good structural quality.

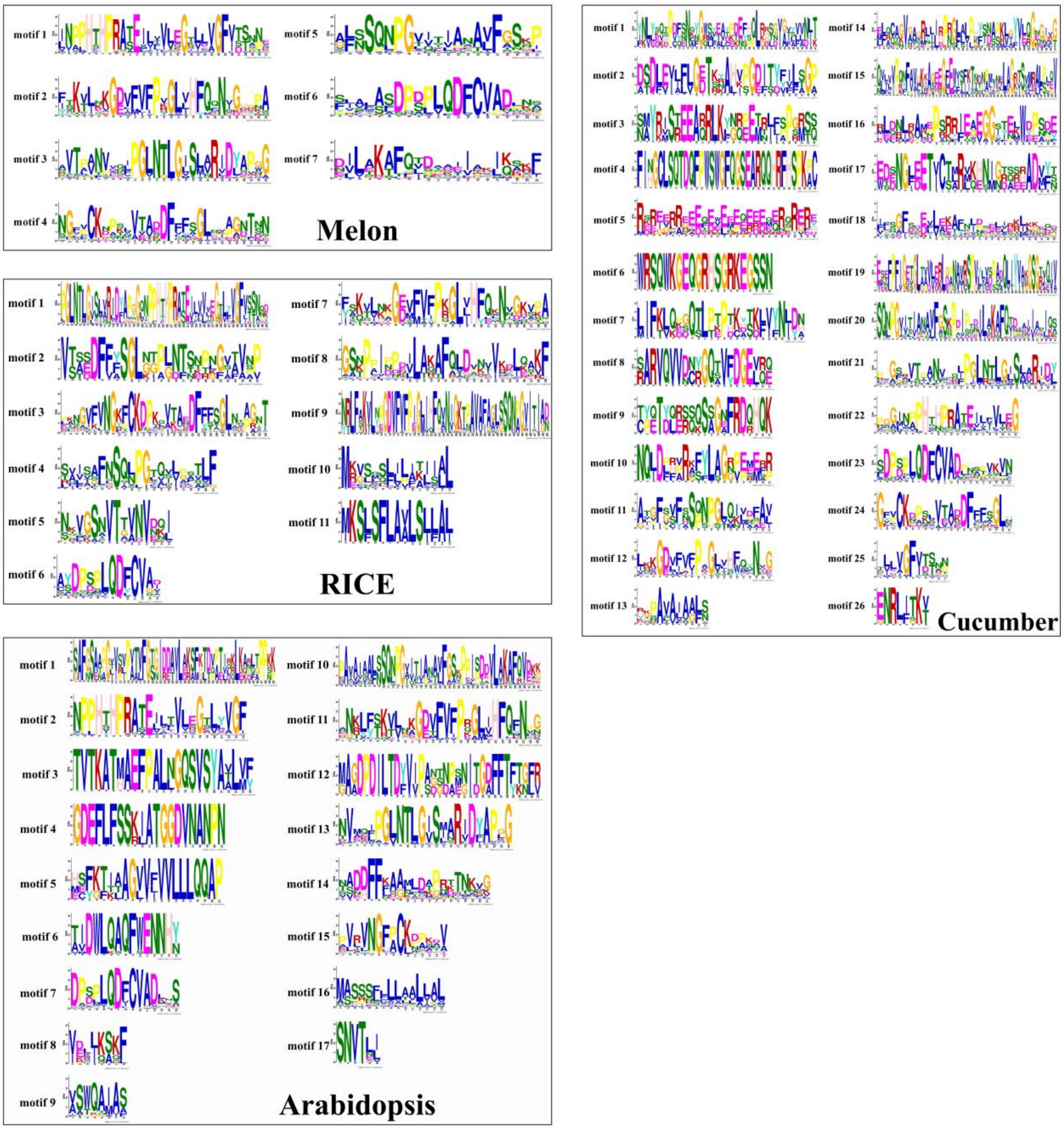

Figure S3. Motifs of melon, rice, Arabidopsis, and cucumber.
